# Polyamine pathways interconnect with GABA metabolic processes to mediate the low-temperature response in plants

**DOI:** 10.3389/fpls.2022.1035414

**Published:** 2022-10-20

**Authors:** Mengyun Xu, Qinwen Yang, Genxiang Bai, Ping Li, Jian Yan

**Affiliations:** ^1^ Key Laboratory of Agro-Environment in the Tropics, Ministry of Agriculture and Rural Affairs, South China Agricultural University, Guangzhou, China; ^2^ Guangdong Provincial Key Laboratory of Eco-Circular Agriculture, South China Agricultural University, Guangzhou, China; ^3^ Guangdong Engineering Research Centre for Modern Eco-Agriculture, South China Agricultural University, Guangzhou, China; ^4^ College of Natural Resources and Environment, South China Agricultural University, Guangzhou, China

**Keywords:** cold stress, polyamines, GABA, ROS, osmolytes

## Abstract

Low temperatures are among the most commonly encountered environmental conditions that adversely affect plant growth and development, leading to substantial reductions in crop productivity. Plants have accordingly evolved coordinated mechanisms that confer low-temperature adaptation and resistance. The plant metabolic network, including polyamines (PAs) and γ-aminobutyric acid (GABA) is reprogrammed to ensure that essential metabolic homeostasis is maintained in response to cold stress conditions. Additionally, GABA might serve as a central molecule in the defense system during low-temperature tolerance in plants. However, our understanding of how these metabolites function in conferring cold tolerance is still far from complete. Here, we summarized how PAs and GABA function in conferring cold tolerance, and describe the crucial role of GABA in the mitigation of ROS during cold stress in plants.

## Introduction

Low-temperature stress has two distinct components: chilling, which is generally defined as lower-than-normal and higher than 0°C growth temperatures, and freezing, which indicates temperatures below 0°C (Raju et al., 2018). The molecular mechanisms underlying low-temperature tolerance have been extensively studied. Several signaling pathways and cold-responsive genes have been characterized and identified in different species, including those from the ICE-CBF-COR transcriptional cascade. Emerging evidence has also indicated that several metabolites, such as polyamines (PAs) and γ-aminobutyric acid (GABA), play pivotal roles in alleviating the damage caused by low temperatures in different plant species (Baier et al., 2019).

## Reprogramming of plant metabolism in response to low temperature

Plants, as sessile organisms, have evolved processes that confer protection against low-temperature conditions. Adaptive processes termed cold acclimation and chilling tolerance, have been developed that enhance tolerance in response to low-temperature exposure, which involves changes in physiological, biochemical, molecular, and metabolic processes (Yadav, 2010). Previous studies have shown that a wide range of metabolites play various roles in low-temperature tolerance, among which particular interest has focused on metabolites such as proline, sugars, secondary metabolites, and polyamines, which can function as osmolytes and are extensively involved in abiotic stress tolerance. Given the importance of such osmolytes in protecting plants against abiotic and biotic stress, they are often collectively referred to as cytoprotectants (Khan et al., 2010).

Plant metabolism responds sensitively and dynamically to low-temperature conditions (Xu and Fu, 2022). With the exposure to temperature stress, plants have developed metabolic modifications that are essential features in response to cold stress (Yadav, 2010; Xu and Fu, 2022). Chilling and/or freezing modify the structure, metabolic properties, and functions of enzymes, as well as the properties of membrane metabolite transporters (Kubien et al., 2003; Yadav, 2010), thereby leading to a diversion of the metabolic flux toward the synthesis of osmoprotectants, including soluble sugars, proline, and polyamines. Therefore, the plant metabolic network, particularly osmoprotectants, must be reprogrammed to ensure that essential metabolic homeostasis is maintained in response to low-temperature conditions.

## Polyamine pathways are interconnected with GABA metabolic processes

Polyamines are aliphatic amines with low molecular mass that play roles in diverse biological processes, and these are mainly present in the free form in higher plants, such as putrescine (Put), spermidine (Spd), and spermine (Spm). Additionally, cadaverine (Cad) and thermospermine (t-Spm), a Spm isomer, are also reported to exist in higher plants (Wang et al., 2019).

**Figure 1:**
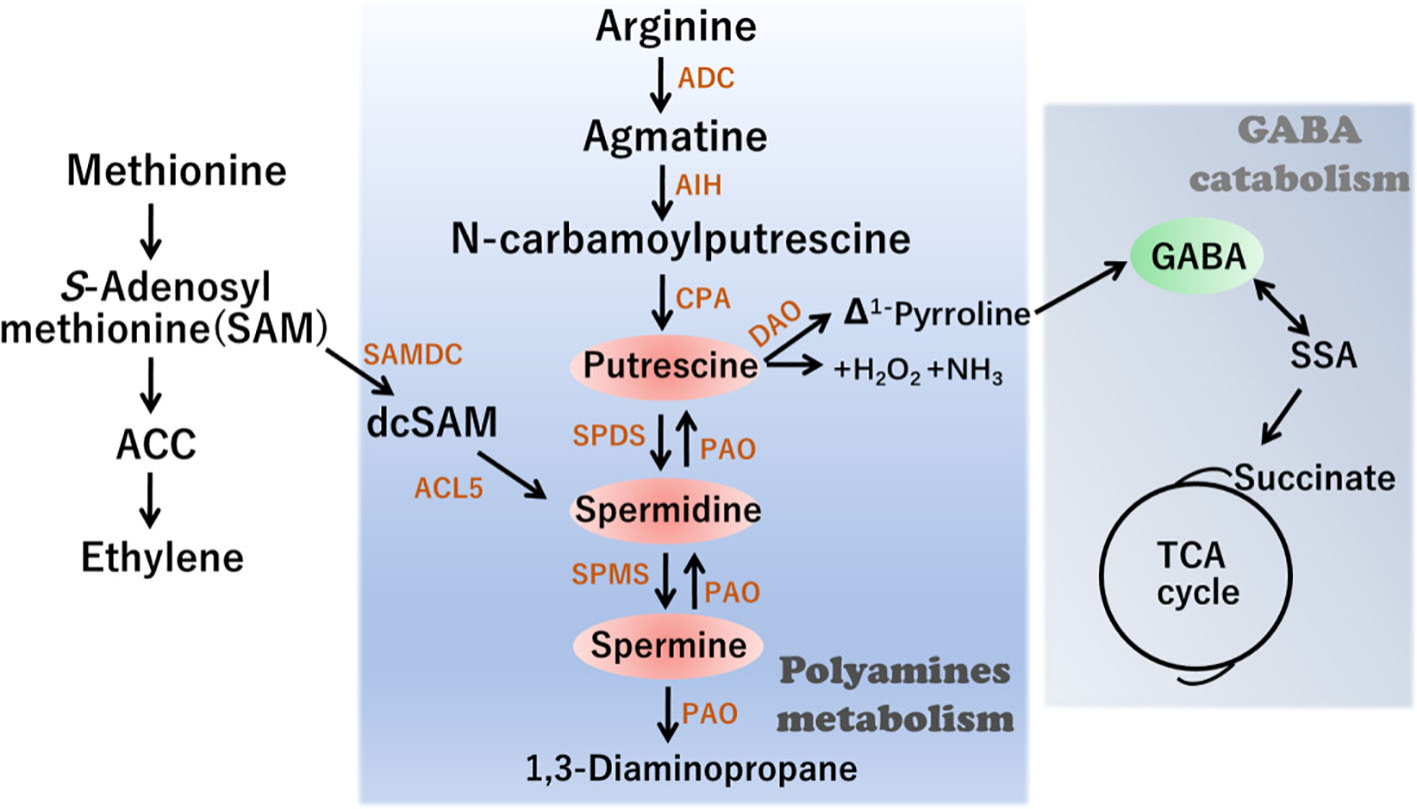
Schematic representation of Polyamines (PAs) metabolism and interconnection with γ-aminobutiric acid (GABA) synthesis. ADC, arginine decarboxylase; AIH, agmatine iminohydrolase; CPA, N-carbamoylputrescine amidohydrolase; DAOdiamine oxidase; PAO polyamine oxidase; SPDS, SPDS, spermidine synthase; SPMS, spermine synthase; ACL5, ACAULIS5; SAMDC, S-adenosylmethionine decarboxylase; ACC, 1-amino-cyclopropane-1-carboxylic-acid.

Polyamine homeostasis is regulated by a dynamic balance among metabolic processes, conjugation, chemical alteration, and transport (Moschou and Roubelakis-Angelakis, 2014; Yu et al., 2019). Given the importance of polyamines, the regulation of their synthesis and accumulation has been well characterized in plants. The polyamine biosynthetic pathway commences primarily with arginine (Arg), which is converted to putrescine *via* three sequential reactions catalyzed by arginine decarboxylase, agmatine iminohydrolase, and N-carbamoylputrescine amidohydrolase (**Figure 1**). Subsequently Spd synthase (SPDS) catalyzes Put conversion to Spd. Finally, Spd is further converted to Spm or T-Spm, two tetraamine isomers, by Spm synthase (SPMS) and T-Spmsynthase, respectively (Hanzawa et al., 2002; Yu et al., 2019). The PA catabolic process is mainly catalyzed by two classes of amine oxidases (AOs): one is a copper-dependent diamine oxidase (DAO) and the other is a flavin adenine dinucleotide (FAD)-dependentpolyamine oxidase (PAO). Notably, putrescine can subsequently be converted into GABA in a reaction catalyzed by DAO. Consequently, the levels of GABA are partly dependent on modifications in polyamine metabolism.

GABA is a four-carbon non-proteinogenic amino acid that acts as a signaling molecule playing multiple roles in a diverse range of organisms. For example, GABA plays a pivotal role in regulating C and N metabolic fluxes by linking amino acid metabolism to the tricarboxylic acid (TCA) cycle (**Figure 1**). Accumulating evidence indicates that GABA is involved in various aspects of plant growth and development, as well as in the biotic/abiotic stress responses in different plant species. Although GABA is generally metabolized *via* the GABA shunt pathway, under stress conditions, it can undergo synthesis *via* a non-enzymatic process involving certain polyamines, including spermidine and putrescine, together with proline (Signorelli et al., 2015; Ansari et al., 2021).

## Role of polyamines in mediating low-temperature tolerance

Plants are sessile organisms that cannot physically escape from stressful environments, including low temperatures, also referred to as cold stress (Pareek et al., 2017). Generally, exposure to cold stress induces a range of physiological and biochemical disturbances, including metabolite imbalance and metabolic dysfunction (Kazemi-Shahandashti and Maali-Amiri, 2018). The production and accumulation of compatible solutes, including polyamines, is a common defense mechanism activated in response to cold stress (Alcázar et al., 2011; Pagter et al., 2017). An alteration in polyamines during the early stage of stress is considered a signal that promotes further signal transduction to activate transcription factors and stress-responsive pathways, such as the ICE-CBF-COR transcriptional cascade, reactive oxygen species (ROS) scavenging, and the antioxidant defense system (Wei et al., 2021; Hwarari et al., 2022).

In plants, polyamines are extensively involved in the responses to abiotic stresses, including low-temperature stress, and the association between polyamines and cold tolerance is well established. Accumulating evidence has revealed that exogenous polyamine treatment enhances tolerance to low temperatures in different plant species. For example, spermidine priming has been demonstrated to enhance polyamine metabolism and hence tolerance to chilling stress in rice (Sheteiwy et al., 2017). Furthermore, spray application of putrescine has been found to substantially reduce chilling injury in peach fruit during storage, regardless of the dose of putrescine applied or the time of application. In contrast, foliar spraying of seedlings with spermidine, spermine, and putrescine is believed to activate a defensive response to enhance cold resistance in winter oilseed rape (Jankovska-Bortkevič et al., 2020). SAM is a precursor not only in PA syntheses but also in ethylene biosynthesis (**Figure 1**), and ethylene was considered as a crucial hormone that plays essential roles in cold stress. For example, ethylene enhanced the cold tolerance *via* the MdERF1B–MdCIbHLH1 regulatory module in apple (Wang et al., 2021)

Genetic manipulation of polyamine biosynthetic genes enhances tolerance to cold stress. Arginine decarboxylase (ADC) is a rate-limiting enzyme that catalyzes the first step of polyamine biosynthesis (Urano et al., 2005). In *Arabidopsis*, exposure to cold stress has been observed to promote increased levels of *AtADC1* and *AtADC2* transcripts. Compared with wild-type plants, mutant plants with T-DNA insertional knockout (*adc1* and *adc2*) of these enzymes were found to accumulate less free putrescine and were more sensitive to freezing. However, the damage caused by freezing conditions could be alleviated by the exogenous application of putrescine (Cuevas et al., 2008). Conversely, by modulating putrescine accumulation, the overexpression of *ADC1* in potatoes has been found to confer a higher level of freezing tolerance (Kou et al., 2018).

## GABA, a key player in mitigating ROS generation during cold stress in plants

In plants, GABA is a ubiquitous four-carbon metabolite and a vital signaling molecule that mediates the responses to biotic and abiotic stress conditions, including pathogen attack, low and high temperature, flooding, drought, soil salinity, and heavy metals (Li et al., 2021). Emerging evidence indicates that GABA participates in the low-temperature regulatory mechanisms of plants. Low temperatures are common unfavorable environmental conditions that limits plant development, leading to significantly reduced plant productivity. Generally, high level of GABA and shunt-related genes are induced in response to low-temperature conditions. For example, compared with non-stressed conditions, a 16-fold increase in GABA levels has been observed in barely seedlings directly exposed to -3°C, and amounts of GABA were also elevated in seedlings exposed to a temperature of -8°C (Mazzucotelli et al., 2006). Further studies in barley have revealed that the accumulation of GABA induces the expression of GABA-shunt genes (Mazzucotelli et al., 2006), thereby providing evidence to indicate the involvement of GABA metabolism in the cold tolerance in plants.

**Figure 2:**
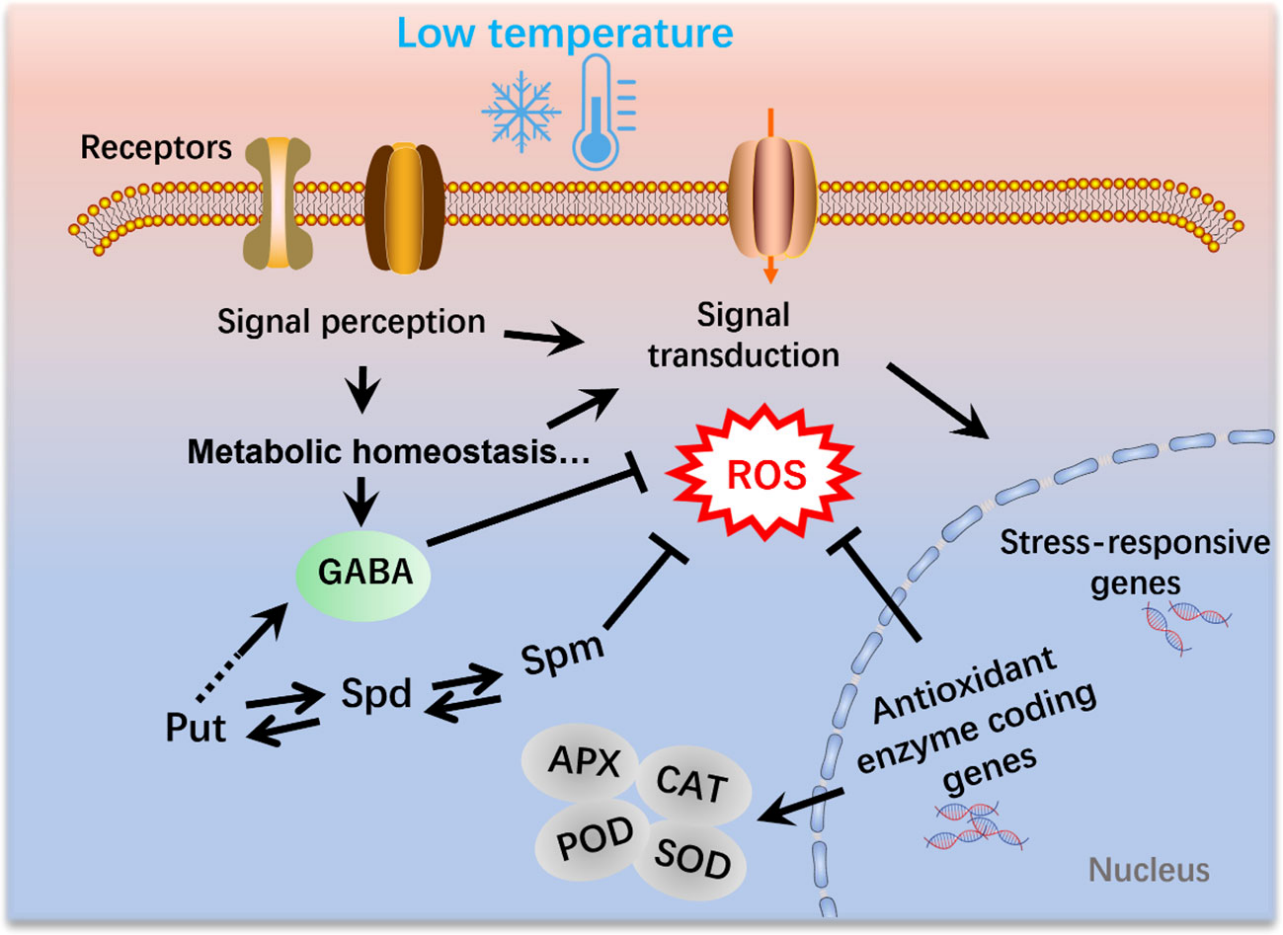
Polyamines and γ-aminobutyric acid (GABA) mediate the mitigation of reactive oxygen species (ROS) production under low-temperature conditions in plants. Cold signals are sensed by receptors and thereby induce a range of physiological, biochemical, and metabolic processes. Moreover, signal transduction leads to the activation of cold-responsive genes. SOD, superoxide dismutase; POD, Peroxidase activity; APX, ascorbate peroxidase; CAT, catalase.

The exogenous application of GABA has been demonstrated to increase GABA levels and enhance cold tolerance in various plant species. In tomato seedlings, for example, accumulation of GABA has been observed in response to chilling treatment. The application of exogenous GABA induces substantially higher amounts of endogenous GABA in tomato seedlings compared with those in control plants. (Malekzadeh et al., 2014). Notably, the antioxidant enzyme activity, malondialdehyde (MDA) and proline displayed significantly decreased level after GABA treatment, whereas the sugar and proline level were significantly enhanced compared to un-treated seedlings (Malekzadeh et al., 2014). Exogenous GABA enhanced the endogenous GABA content by increasing the expression of the glutamate decarboxylase (GAD) gene and decreasing GABA transaminase (GABA-T) gene level (Shekari et al., 2021). In most cases, the application of GABA at low temperatures has been associated with the activation of the antioxidant defense system. For example, Wang et al. (2014) revealed that the application of GABA alleviated chilling injury in banana fruit by promoting proline accumulation and enhancing antioxidant capacity. Similarly, the application of GABA during the reproductive stage in tomato plants has been found to substantially alleviate chilling-induced oxidative damage by enhancing the activity of CAT, SOD and APX (Abd Elbar et al., 2021), thereby tending to indicate that GABA enhances the tolerance to low-temperature stress by modulating ROS content (**Figure 2**).

The generation of ROS is an inevitable consequence of plant aerobic metabolism, which occurs in multiple cellular compartments, including the chloroplasts, mitochondria, apoplasts, and peroxisomes (Gill and Tuteja, 2010). Emerging evidence has indicated that the role of ROS in plants might be a double-edged sword, in that ROS also act as vital signal molecules involved in plant growth and development, particularly in response to abiotic and biotic stresses, including drought, salinity, metal toxicity, heat shock, and cold stress (Bailey-Serres and Mittler, 2006; Gapper and Dolan, 2006; Waszczak et al., 2018; Hasanuzzaman et al., 2020). GABA serves as a central molecule in the defense system of plants and is extensively involved in the mitigation of ROS in response to different stresses, including exposure to low temperatures (Ansari et al., 2021). The GABA shunt pathway effectively bypasses two enzymes in the TCA cycle that have been established to be sensitive to oxidative stress (Nicolas and Hillel, 2004; Janse van Rensburg and Van den Ende, 2020). Moreover, many GABA-related components have been demonstrated to play vital roles in ROS scavenging and detoxification under stress conditions (Jalil et al., 2017; Ansari et al., 2021). For example, succinic semialdehyde dehydrogenase (SSADH), a GABA shunt enzyme, catalyzes the conversion of succinic semialdehyde to succinate in the mitochondria. Previous studies have shown that SSADH is essential for ROS homeostasis in plants (Bao et al., 2015). Given that the application of GABA under low-temperature conditions generally modulates the activities of oxidative enzymes, suggests that GABA plays a crucial role in the mitigation of ROS during cold stress in plants (**Figure 2**).

## Concluding remarks

Although the involvement of several key metabolites, including polyamines and GABA, has been demonstrated during the low-temperature stress response of plants, a detailed understanding of how metabolites participated in cold tolerance is imperative. Further studies are needed to gain a better understanding of the pathways and regulatory networks of key metabolites. Additional effort should also be devoted to elucidating the dynamics of these metabolites in the development of low-temperature tolerance. In addition, the rapid development of metabolomics technology in plants will provide new opportunities to identify novel and/or unknown metabolites associated with the response to low-temperature conditions. We believe that with further in-depth research on the mechanisms of key metabolites involved in low-temperature responses, the knowledge thus gained will make it possible to enhance the cold tolerance and productivity of plants.

## Author contributions

MX, QY, and JY undertook the literature review and wrote the manuscript. GB and PL edited the manuscript. All authors contributed to the article and approved the submitted version.

## Funding

This manuscript was funded by the National Natural Science Foundation of China (31970370, 32270417), jointly supported by the Science and Technology Project of Nujiang Prefecture, Yunnan Province, China (2020CY004) and the Science and Technology Planning Project of Guangdong Province, China (2019B030301007).

## Conflict of interest

The authors declare that the research was conducted in the absence of any commercial or financial relationships that could be construed as a potential conflict of interest.

## Publisher’s note

All claims expressed in this article are solely those of the authors and do not necessarily represent those of their affiliated organizations, or those of the publisher, the editors and the reviewers. Any product that may be evaluated in this article, or claim that may be made by its manufacturer, is not guaranteed or endorsed by the publisher.
